# Syndrome-oriented CNS infectious complications after hematopoietic stem-cell transplantation and CAR T-cell therapy, part 1—viral encephalitis, meningitis, and myelitis: recommendations from the Practice Harmonization & Guidelines Committee of the EBMT

**DOI:** 10.1016/j.eclinm.2026.104312

**Published:** 2026-11-05

**Authors:** José Luis Piñana, Simone Cesaro, Jan Styczynski, Martin Schmidt-Hieber, Malgorzata Mikulska, Dionysios Neofytos, Francesco Baccelli, David Navarro, Francesco Onida, Annalisa Ruggeri, Isabel Sanchez-Ortega, Rafael de la Camara, Dina Averbuch, Ibrahim Yakoub-Agha, Per Ljungman

**Affiliations:** a Department of Hematology, Hospital Clínico Universitario de Valencia, INCLIVA, Biomedical Research Institute, Valencia, Spain; b EBMT Infectious Disease Working Party, Leiden, the Netherlands; c Pediatric Hematology Oncology, Department of Mother and Child, Azienda Ospedaliera Universitaria Integrata Verona, Italy; d Department of Pediatric Hematology, Oncology, Immunology and Transplantology, Nicolaus Copernicus University Torun UMK, Jurasz University Hospital, Collegium Medicum, Bydgoszcz, Poland; e Med. Clinic (Hematology, Oncology, Pneumology, Nephrology and Diabetology), Medical University Lausitz - Carl Thiem (MUL - CT), Thiemstrasse 111, 03048, Cottbus, Germany; f Division of Infectious Diseases, Department of Health Sciences (DISSAL), University of Genova, Genova, Italy; g IRCCS Ospedale Policlinico San Martino, Genova, Italy; h Division of Infectious Diseases, University Hospital of Geneva, Geneva, Switzerland; i Pediatric Hematology and Oncology, IRCCS Azienda Ospedaliero-Universitaria di Bologna, Bologna, Italy; j Department of Microbiology, Hospital Clínico Universitario of Valencia, INCLIVA, Biomedical Research Institute, University of Valencia, Valencia, Spain; k Department of Microbiology, School of Medicine, University of Valencia, Valencia, Spain; l Hematology and BMT Centre, ASST Fatebenefratelli-Sacco, University of Milan, Milan, Italy; m Practice Harmonization and Guidelines Committee of the EBMT, Barcelona, Spain; n Haematology and BMT Unit, IRCCS San Raffaele Hospital, Milan, Italy; o EBMT Executive Office, Barcelona, Spain; p Hematology División, Hospital de la Princesa, Madrid, Spain; q Faculty of Medicine, Hebrew University of Jerusalem, Jerusalem, Israel; r Hadassah Medical Center, Jerusalem, Israel; s CHU de Lille, Univ Lille, INSERM U1286, Infinite, 59000, Lille, France; t Department of Cellular Therapy and Allogeneic Stem Cell Transplantation, Comprehensive Cancer Center, Department of Infectious Diseases, Karolinska University Hospital, Stockholm, Sweden; u Department of Medicine Huddinge, Karolinska Institutet (KI), Stockholm, Sweden

**Keywords:** Allogeneic hematopoietic cell transplantation, Chimeric antigen receptor T cell therapy, Viral encephalitis, Viral meningitis, Viral myelitis, Harmonization and best practice EBMT recommendations

## Abstract

Viral encephalitis, meningitis, and myelitis are rare but life-threatening complications after autologous or allogeneic hematopoietic cell transplantation (HSCT) and chimeric antigen receptor (CAR) T-cell therapy, associated with 30–60% mortality and frequent neurological sequelae. The absence of harmonised definitions and diagnostic criteria contributes to delayed diagnosis, underreporting, and substantial heterogeneity across centres. This EBMT Practice Harmonization and Guidelines Committee expert consensus provides practical recommendations for the definition, diagnosis, treatment, response assessment, and prevention of viral encephalitis/meningitis and viral myelitis, developed through systematic literature review and multidisciplinary expert consensus following EBMT methodology. We propose standardised diagnostic categories for proven and probable viral encephalitis/meningitis and myelitis, alongside a unified diagnostic algorithm integrating clinical assessment, neuroimaging, electroencephalography, cerebrospinal fluid analysis, and targeted virological testing. We highlight key neurotropic viruses for inclusion in initial cerebrospinal fluid multiplex PCR panels, including human herpesvirus 6, herpes simplex virus 1/2, varicella zoster virus, cytomegalovirus, Epstein-Barr virus, adenovirus, polyomaviruses BK/JC, and selected community-acquired respiratory viruses, such as EV71, and recommend prompt, risk-based empirical antiviral therapy in suspected cases. These recommendations aim to standardise clinical practice, facilitate multicenter research, and improve outcomes. However, many rely on limited observational evidence or expert opinion, underscoring the need for prospective studies.

## Introduction

Viral encephalitis, meningitis, and myelitis represent a rare but potentially devastating complication after allogeneic hematopoietic cell transplantation and chimeric antigen receptor (CAR) T-cell therapy. While the global incidence of viral encephalitis in the general population is approximately 5–10 per 100,000 persons per year,^1^, ^2^, ^3^ its incidence and case-fatality rates are markedly higher in immunocompromised patients.^4^, ^5^, ^6^ Profound and prolonged T-cell dysfunction, disruption of the blood–brain barrier, and intensive immunosuppressive therapy predispose to viral invasion of the central nervous system (CNS).^6^, ^7^ More than 100 viruses can cause viral encephalitis/meningitis or myelitis,^8^ but this document focuses on those most commonly reported in cell therapy recipients.

To date, standardised definitions, diagnostic algorithms, and management strategies for viral encephalitis/meningitis and myelitis in these immunocompromised populations remain lacking, contributing in part to under-recognition, delayed diagnosis, and reporting heterogeneity across studies. Initiated by EBMT Infectious Diseases Working Party (IDWP) and Practice Harmonization and Guidelines Committee (PH&G), the document provides a holistic, syndrome-oriented approach to viral CNS infections after autologous or allogeneic HSCT and CAR T-cell therapy, encompassing encephalitis, meningitis, and myelitis. It harmonises definitions, diagnosis, treatment principles, and response evaluation. Encephalitis and meningitis are addressed together because they are closely related and may coexist as meningoencephalitis. Evidence derives predominantly from allogeneic HSCT recipients; extrapolation to autologous HSCT and CAR T cells requires the consideration of the different types of immunosuppression given to these patient groups. Given its rarity and limited evidence, viral myelitis is addressed separately, covering recognition, differential diagnosis, diagnostic evaluation, and management.

## Methods

### Search strategy and selection criteria

A structured literature search was conducted in PubMed to identify relevant studies published between January 1, 2000, and September 2025. The strategy combined Medical Subject Headings (MeSH) and free-text terms related to viral CNS infections, including “viral encephalitis,” “viral meningitis,” “meningoencephalitis,” “viral myelitis,” “encephalomyelitis,” and “central nervous system infection,” with transplantation-related terms: “Hematopoietic Stem Cell Transplantation,” “Bone Marrow Transplantation,” “Transplantation, Autologous,” “Transplantation, Allogeneic,” “stem cell transplant⁎,” “bone marrow transplant⁎,” “hematopoietic cell transplant⁎,” “HSCT,” “BMT,” “CAR-T⁎,” and “transplant recipient⁎.” Additional targeted searches combined CNS-related terms with specific viral pathogens, including herpes simplex virus, varicella-zoster virus, cytomegalovirus, human herpesvirus 6, Epstein–Barr virus, adenovirus, enterovirus, JC and BK polyomaviruses, community-acquired respiratory viruses, and arboviruses. Searches were limited to human studies published in English. Given the rarity of these complications, case reports and small case series were eligible. Reference lists of relevant publications and guidelines were manually reviewed to identify additional studies.

Definitions were aligned with the International Encephalitis Consortium criteria^9^ and refined for allogeneic HSCT and CAR T-cell populations.

### EBMT workshop and consensus

This harmonization project includes two companion manuscripts (Part 1 of a syndrome-oriented series on CNS infectious complications following HSCT and CAR T-cell therapy, focusing on viral etiologies, and Part 2 will address bacterial, fungal, and parasitic CNS infections) that were developed as a single project following the expert-based methodology of the EBMT PH&G Committee, as previously described by Yakoub-Agha et al.^10^ The intended use is for specialists in the field managing these types of patients with the aim of supporting clinical decisions.

A multidisciplinary panel of hematology (n = 4), infectious diseases (n = 3), microbiology (n = 1), pediatric specialists (n = 4), and members of the EBMT PH&G Committee (n = 4) was convened. Committee members were selected and invited based on their clinical and scientific expertise in the field and the involvement in dedicated IDWP subcommittees, with consideration given to geographic diversity and representation of complementary specialties and competencies. Before the workshop, predefined clinical questions were formulated, and a structured literature review of the published registry analyses, cohort studies, case series, and systematic reviews were conducted. Designated panel members reviewed and summarised the evidence relevant to each question and circulated it to the group. Panel members discussed the evidence and proposed recommendations during a two-day EBMT workshop. Panel members presented and discussed available evidence and preliminary recommendations in plenary sessions. Given the scarcity and heterogeneity of the available evidence in the targeted patients’ population, particularly the absence of randomised trials or comparative observational studies for most diagnostic and therapeutic interventions, and the frequent need to extrapolate from other populations, we elected not to formally grade the evidence or the strength of recommendations. Instead, the supporting evidence and its limitations were described narratively. Recommendations based predominantly on indirect evidence, good clinical practice, or multidisciplinary expert consensus were explicitly formulated, and their wording was adjusted to reflect the associated uncertainty. Consensus was reached through iterative discussion and review of the relevant literature, with disagreements addressed and recommendations revised until all panel members were in agreement. The final recommendations were unanimously approved by the panel before internal review and validation in accordance with the EBMT PH&G Committee process in 2025.

## Viral encephalitis

### Epidemiology, risk factors and viral characteristics

Overall, viral encephalitis incidence after allogeneic HSCT ranges from 0.5% to 1.5%.^11^, ^12^, ^13^, ^14^ Most common viruses include Human herpes virus-6 (HHV-6), human herpes simplex virus types 1–2 (HSV I-II), cytomegalovirus (CMV), Epstein–Barr virus (EBV), varicella-zoster virus (VZV), adenovirus, JC polyomavirus, Enterovirus 71 (EV71).^11^, ^12^, ^13^, ^15^, ^16^, ^17^ Of note, in allogeneic HSCT and CAR T-cell recipients, viral encephalitis most commonly results from reactivation of latent viruses under profound immunosuppression. Overall incidence and main characteristics of viral encephalitis in allogeneic HSCT recipients by virus type are summarised in Table 1. All viral encephalitis, except for HHV-6, are rare to very rare. Information on viral myelitis is even more limited.^22^, ^23^, ^24^ Viral encephalitis in CAR T-cell recipients is also very rarely reported and limited to case series,^25^, ^26^ even with systematic cerebrospinal fluid (CSF) evaluation.^27^ However, it has been increasingly recognised, particularly due to HHV-6 and JC polyomavirus in the setting of delayed immune reconstitution.^28^, ^29^, ^30^, ^31^, ^32^, ^33^ HHV-6 reactivation or detection has been reported during ex vivo T-cell activation and expansion for manufacturing of CAR T cells. However, these observations do not demonstrate transmission through the infused CAR T-cell product or establish an association with post-infusion HHV-6 encephalitis.^34^

**Table 1:** Incidence and most common pathogen involved in viral encephalitis/meningitis after allogeneic HCT.

Pathogen	Approximate overall incidence after allo-HCT	Peak onset after allo-HCT days	High-risk transplant settings	Classical clinical or MRI pattern	References
HHV-6	0.45–2.0% overall; up to 11% after cord-blood transplantation	+20 to +40	Cord blood, haploidentical, T-cell–depleted grafts	Limbic encephalitis with bilateral hippocampal hyperintensity	11, 12, 13, 14, 18, 19
CMV	0.1–0.3%	+30 to +100	T-cell–depleted, GVHD, high-dose steroids	Ventriculitis, periventricular/ependymal lesions	16, 19
HSV-1/2	<0.1%	Early (<+30)	Profound neutropenia, mucositis, steroid exposure	Temporal lobe necrotising lesions	12, 13, 19
VZV	0.1–0.3%	Late (>+100)	Chronic GVHD, prolonged immunosuppression	Multifocal vasculitic or ischemic lesions	4, 12, 13, 18, 19
EBV	<0.2%	Variable	PTLD, rituximab-naïve, delayed B-cell recovery	Diffuse or limbic lesions; PTLD-associated CNS mass	12, 20
Adenovirus (AdV)	<0.05%	+20 to +60	Cord blood, pediatric, severe lymphopenia	Diffuse or basal ganglia lesions; ventriculitis	12, 15
Polyomaviruses (JC/BK)	<0.05%	Late (>+180)	Severe T-cell deficiency, rituximab, chronic GVHD	PML pattern (asymmetric white-matter lesions)	13, 18, 21

Abbreviations: allo-HCT, allogeneic hematopoietic stem cell transplantation; HHV-6, human herpesvirus 6; CMV, cytomegalovirus; HSV-1/2, herpes simplex virus type 1 or 2; VZV, varicella zoster virus; EBV, Epstein–Barr virus; PTLD, post-transplant lymphoproliferative disorder; AdV, adenovirus; JC/BK, JC or BK polyomavirus; PML, progressive multifocal leukoencephalopathy; CARVs, community-acquired respiratory viruses; EV71, enterovirus 71; RSV, respiratory syncytial virus; GVHD, graft-versus-host disease; CNS, central nervous system.

Viral encephalitis development after allogeneic HSCT results from a complex interplay between transplant-related factors, immunosuppressive exposure, virological dynamics, and host immune reconstitution. Understanding these risk profiles is essential for early suspicion, diagnosis, and intervention. Table 2 summarises the most commonly reported risk factors for individual viruses and immunosuppression conditions.

**Table 2:** Summary of key characteristics and/or risk factors by category and by pathogen.

Category	Specific factors	Associated viruses	Comments	References
Transplant-related	Cord blood transplantation (CBT)	HHV-6 (incidence up to 11%)	Delayed T-cell reconstitution; high early viral reactivation rates	4, 11, 12, 13, 14, 35, 36
	Haploidentical or mismatched donor	HHV-6, CMV, AdV	Intense T-cell depletion and delayed immune recovery	11, 15, 16
	T-cell–depleted grafts (in vivo or ex vivo)	HHV-6, CMV, AdV, JC	Reduced antiviral T-cell immunity	13, 16, 37
	Early post-transplant phase (≤+100 days)	HHV-6	Period of profound lymphopenia and immunosuppression	4, 11, 12, 13, 14, 38
	Late post-transplant phase (>+100 days)	VZV, CMV, HSV, AdV, JC, EBV	Immune exhaustion, chronic GVHD, long-term immunosuppression	4, 12, 13, 16, 37
Host-related	Age <30 years, pediatric age	HHV-6	Immature immune system, higher reactivation rates	13, 36
	Profound lymphopenia (CD4, CD8, NK)	HHV-6, CMV, EBV, AdV, JC	Impaired cellular immune control	4, 16, 36, 39
Immunosuppressive therapy	Alemtuzumab, ATG, OKT3, T-cell depletion	HHV-6, EBV, AdV, JC	Profound T- and B-cell depletion; delayed immune recovery	4, 13, 40
	Including mycophenolate mofetil as GvHD prophylaxis	HHV-6 (neurotoxicity mimicry)	Increased risk of GVHD and delayed T-cell reconstitution	^35^
Virological factors	High plasma viral load	HHV-6, CMV, AdV	Marker of uncontrolled viral replication and CNS tropism	4, 11, 41, 42
	Coinfection or viral co-reactivation	HHV-6 + CMV, EBV, AdV	Synergistic immunosuppression and delayed clearance	16, 43
Post-transplant complications	Severe acute or chronic GVHD	HHV-6, JC, CMV, EBV, AdV, VZV	Prolonged immunosuppression; disrupted CNS immune surveillance	4, 14, 36, 38, 41, 43
	Engraftment syndrome, high IL6 levels	HHV-6	Linked to cytokine dysregulation and impaired antiviral defence	35, 42, 43
	Corticosteroids (≥1 mg/kg prednisolone equivalent)	CMV, VZV, AdV, HHV-6	Lymphocyte apoptosis; viral replication enhancement	38, 44
CAR-T therapy–specific	Tocilizumab/corticosteroids for concurrent ICANS	HHV-6, JC	Diagnostic overlap; corticosteroid use may exacerbate viral risk	28, 30, 31, 33, 34
	Prolonged cytopenias (>30 days), B-cell aplasia and hypogammaglobulinemia	JC	Loss of neutralising antibody activity	30, 31, 33, 34
	BCMA product, prior allo-HCT	CMV	profound immunosuppression status	^29^

Abbreviations: HHV-6, human herpesvirus 6; AdV, adenovirus; CMV, cytomegalovirus; JC, JC polyomavirus; HSV, herpes simplex virus; VZV, varicella zoster virus; EBV, Epstein–Barr virus; CARVs, community-acquired respiratory viruses; CD4, cluster of differentiation 4; GVHD, graft-versus-host disease; CNS, central nervous system; CBT, cord blood transplantation; CAR-T, chimeric antigen receptor T-cell therapy; ICANS, immune effector cell–associated neurotoxicity syndrome; ATG, anti-thymocyte globulin; OKT3, muromonab-CD3.

HHV-6B is the leading cause of viral encephalitis after allogeneic HSCT, accounting for 70–80% of reported cases.^12^, ^13^, ^14^ The EBMT IDWP analysis estimated an overall incidence of 0.45%, with higher rates in cord-blood (2.14%), unrelated donor (0.68%), and haploidentical (0.51%) transplants than in matched sibling donor transplants (0.06%).^11^ Key risk factors include cord blood, mismatched or haploidentical grafts, T-cell depletion, younger age, and high HHV-6 viremia.^11^, ^35^, ^36^, ^38^, ^40^, ^41^, ^42^ Symptoms typically appear around 20–40 days after transplantation and include confusion, seizures, and amnesia.^12^

CMV is a rare cause of viral encephalitis after allogeneic HSCT but carries a high mortality (up to 70%). Most of the data come from the pre-letermovir era, and letermovir prophylaxis has decreased all forms of CMV disease.^16^, ^45^ While CMV viral encephalitis MRI commonly shows periventricular enhancement or ependymal involvement, these abnormalities may be absent in a significant percentage of patients.^46^

HSV I-II encephalitis usually presents early in the post-transplantation period, particularly in the absence of prophylaxis^12^, ^13^ or in cases of acyclovir-resistant HSV infection.^47^

VZV CNS infection typically manifests later in patients with chronic graft-versus-host disease (GVHD) and prolonged corticosteroid exposure, presenting with vasculitis or ischemic lesions.^4^, ^12^, ^13^ VZV viral encephalitis MRI may show encephalitic changes, but may also appear normal.^48^

EBV encephalitis or EBV encephalomyelitis may occur with or without post-transplant lymphoproliferative disorder (PTLD). It generally presents months after transplantation, often in rituximab-naïve or heavily immunosuppressed patients.^12^, ^49^, ^50^, ^51^

Only a few cases of adenovirus encephalitis have been reported^12^ in pediatric or cord-blood transplant recipients early after transplantation. Disseminated disease often coexists, and prognosis remains poor.^12^, ^15^

JC virus–associated PML typically occurs late in recipients with chronic GVHD, rituximab exposure, or profound T-cell lymphopenia.^13^, ^39^ In an EBMT series of 23 PML cases, classified following American Academy of Neurology criteria,^21^ the median onset was 14 months after transplantation, with 78% mortality despite treatment.^37^ BK virus encephalitis has only been reported in isolated allogeneic HSCT cases.^12^

#### Other viruses that rarely cause viral encephalitis after allo-HSCT

Non-herpesvirus viral encephalitis after allogeneic-HSCT is limited to isolated reports. Community-acquired respiratory viruses (CARVs), including influenza,^52^ RSV,^43^ and EV,^53^ have been detected in CSF, often in patients with hypogammaglobulinemia. EV accounted for 1.4% of viral encephalitis cases, with median onset at day +121 and 33.3% 6-month mortality.^54^ SARS-CoV-2 has been associated with encephalitis-like syndromes, though CSF detection and direct CNS involvement remain unproven. Parvovirus B19 and Coxsackie viruses (particularly CoxB3) caused 3.3% and 1.4% of viral encephalitis cases, respectively, with late onset and high mortality for Coxsackie.^12^, ^54^ HHV-7 has been linked to fatal viral encephalitis in two cases.^55^ Flaviviruses, including WNV,^56^ JEV, ZIKV, TBEV, and Tuscany virus, are potential but anecdotal causes in this setting.

## Viral meningitis without encephalitis

Isolated viral meningitis, defined as meningeal inflammation without parenchymal brain involvement, is rarely reported in allogeneic HSCT or CAR T-cell recipients and has not been independently quantified in published series.^4^, ^12^, ^57^ Instead, most viral CNS infections appear to occur along a continuum of meningoencephalitis, with encephalopathic features dominating the clinical presentation and CSF abnormalities often being subtle or absent.^4^, ^57^ Non-polio enteroviruses, including echoviruses and coxsackieviruses; HSV-2; and, in children, human parechovirus are well-recognised causes of meningitis in the general population, whereas EV71 more commonly causes rhombencephalitis. In immunocompromised patients, herpesviruses such as HHV-6 and HSV-1 are more typically present with encephalitic features.^57^ Syndromic CSF PCR panels may also detect viral pathogens in patients without overt encephalitis, particularly during the early stages of infection or in milder cases. Although many cases resolve with supportive care, antiviral treatment, usually with i. v. acyclovir, should be given for HSV-2 or VZV meningitis.

## Harmonised definitions

### Purpose and scope

The following harmonised definitions were established to enable uniform case classification, reporting, and outcome assessment of viral encephalitis/myelitis in recipients of allogeneic HSCT or CAR T-cell therapy. Each definition is structured to allow grading of diagnostic certainty (proven, probable) and to incorporate objective parameters for assessing treatment response, thereby supporting either diagnostic, clinical management, or research comparability.

### General definition

Encephalitis is defined as inflammation of the brain parenchyma associated with neurological dysfunction.^9^ viral encephalitis refers here to direct viral infection of the CNS, whereas post-infectious or immune-mediated encephalitis constitutes a distinct entity. Clinical manifestations may include altered mental status, cognitive or behavioural changes, seizures, and focal or multifocal neurological deficits, with or without fever, and an acute, subacute, or chronic course. Encephalopathy may present similarly but lacks confirmed brain inflammation and may result from metabolic, toxic, drug-related, infectious, or other causes.^9^ Although encephalopathy may prompt investigation for viral encephalitis, it is a presenting neurological syndrome rather than diagnostic evidence of encephalitis. In profoundly immunosuppressed patients, however, attenuated inflammatory responses may result in absent CSF pleocytosis or characteristic neuroimaging abnormalities despite genuine CNS infection. Viral meningeal involvement is frequently closely linked to encephalitis, both clinically and diagnostically, and both may coexist as meningoencephalitis. CSF examination, neuroimaging and virological testing commonly form part of the same diagnostic assessment.

### Diagnostic classification

To harmonise diagnostic certainty across EBMT centres, cases are categorised as proven or probable viral encephalitis and proven viral meningitis based on clinical and virological evidence, as summarised in Table 3. Clinicians often rely on surrogate indicators such as CSF inflammation or parenchymal changes on neuroimaging to infer viral encephalitis/meningitis. Nevertheless, viral encephalitis/meningitis may still be present even without marked CSF pleocytosis or identifiable abnormalities on imaging studies; therefore, if these parameters are absent, they do not conclusively exclude proven or probable viral encephalitis. CMV-related viral encephalitis has been addressed in the recently published ECIL-10 guidelines.^58^

**Table 3:** Summary of viral encephalitis/meningitis and myelitis definitions according to the certainty that the neurological syndrome is caused by a virus.

Entity	Viral encephalitis	Viral meningitis	Myelitis
Definitions	VE is defined based on the presence of compatible neurological syndrome^a^, radiological image of encephalitis (CT or, preferably MRI), and evidence of proven or probable viral origin	Viral meningitis is defined based on the presence of meningitis, defined as relevant clinical presentation and evidence of meningeal inflammation^c^ and proven viral origin	Viral myelitis is defined based on clinical manifestations and evidence of proven or probable viral origin
Relevant clinical presentation	Altered mental status ≥24 h ± seizures, acute cognitive dysfunction, behavioural changes, and/or multifocal or focal neurological deficits	Symptoms may vary from mild to severe and include the following: fever and/or signs and symptoms of CNS disorder, such as vomiting, headache, photophobia, irritability, confusion, mental status changes, seizures, lethargy, nuchal rigidity, or other signs of meningeal irritation. In infants, it can include bulging fontanelle, irritability, poor feeding, respiratory distress, pale or marble skin, and hyper- or hypotonia	Myelitis may present with acute limb weakness or flaccid paralysis, hyporeflexia or areflexia, gait impairment, sensory disturbances—including paresthesia, neuropathic pain, and a band-like truncal sensation—and bladder or bowel dysfunction. In severe cases, involvement of upper spinal cord segments or cranial nerves may cause respiratory failure, facial weakness, dysarthria, or dysphagia.
Proven infectious origin	Virus detected by PCR or NGS in brain or histopathologic evidence of viral inclusions IHC or EM.	Detection of a plausible causative virus in CSF by PCR^b^, NGS or viral culture	Detection of a plausible causative virus in CSF by PCR^b^, NGS or culture, or demonstration of the virus in spinal cord tissue by molecular testing, culture, IHC or in situ hybridisation
Probable infectious origin	Virus detected by PCR/NGS in CSF without tissue confirmation.	Not relevant^d^	Temporally associated detection of a plausible virus in blood or another relevant site, when virus detection in CSF or spinal cord tissue is not present with reasonable exclusion of compressive, vascular, malignant, toxic-metabolic and immune-mediated causes

Abbreviations, MRI, means magnetic resonance imaging; EEG, electroencephalogram; PCR, polymerase chain reaction; NGS, next-generation sequencing; IHC, immunohistochemistry; EM, electron microscopy; CSF, cerebrospinal fluid.

a Compatible neurological syndrome refers to symptoms due to acute or subacute temporal relationship between neurological symptom onset and the suspected viral infection, reasonable exclusion of alternative causes, and supportive objective evidence of CNS involvement, such as inflammatory CSF abnormalities, compatible neuroimaging findings, or relevant EEG abnormalities.

b Detection of viral nucleic acid in CSF should not be interpreted as causal in isolation. Classification requires temporal and biological coherence with the neurological syndrome, reasonable exclusion of alternative diagnoses, and supporting evidence of CNS involvement. In profoundly immunosuppressed patients, the absence of CSF pleocytosis or characteristic neuroimaging abnormalities does not necessarily exclude viral encephalitis. For HHV-6, CMV and EBV, incidental detection, systemic reactivation, blood contamination, and, for HHV-6, chromosomal integration should be considered.

c Meningeal inflammation is defined as the presence of at least one of the following: (a) abnormal CSF analysis with pleocytosis (>5 white cells/μLin adults or above age-related norms in children) (b) meningeal enhancement compatible with meningitis on contrast-enhanced brain CT or MRI (if lumbar puncture was not performed recently, as lumbar puncture can cause a non-specific meningeal enhancement). In severely immunocompromised patients (e.g., profound neutropenia <100 cells/mm3), the signs of inflammation (pleocytosis or imaging findings) can be attenuated. Case-by-case discussion is needed when a patient with suspected meningitis and identification of the plausible pathogen in the non-bloody CSF presents with a complete absence of meningeal inflammation.

d In the absence of direct viral identification in CSF, NGS, or culture, such cases cannot be reliably attributed to a viral etiology and are therefore classified as meningitis of unknown etiology.

### Relevant considerations

The possibility of a traumatic lumbar puncture and contamination by peripheral blood should be assessed based on the CSF appearance and the red blood cell (RBC) count. When interpreting RBC/white blood cell (WBC) ratios (in cases where there are large numbers of RBC in the CSF, such as with a traumatic lumbar puncture), the following formula may allow correction of the WBC count: True CSF WBC = actual CSF WBC - (WBC in blood x RBC in CSF)/RBC in blood.^59^ Notably, rules for adjusting leukocytes in blood-contaminated CSF have not been well validated.

In probable cases, causality should be attributed to the identified virus only when temporal, clinical, and radiologic correlations are consistent, and alternative etiologies (drug toxicity, GVHD, transplant-associated microangiopathy or ICANS) have been carefully evaluated and judged to be unlikely.

In CAR T-cell recipients, the possible diagnosis and management of ICANS should be prioritised when timing and phenotype are typical, particularly with previous CRS, without delaying indicated infectious evaluation or empirical antivirals. Given its low yield in typical ICANS,^60^ reserve lumbar puncture for atypical timing/evolution, infectious features, suggestive MRI/EEG or virology, or poor clinical response to treatment.

## Harmonised diagnostic algorithm

Diagnosing viral encephalitis after allogeneic HSCT or CAR T-cell therapy is complex due to overlapping syndromes such as ICANS or drug-induced neurotoxicity,^18^, ^61^, ^62^ non-specific or delayed CSF findings, and limited sensitivity of early virological testing. Fig. 1 outlines the proposed EBMT diagnostic algorithm, structured into six key steps: (1) clinical suspicion; (2) biochemical screening; (3) brain MRI with pathogen-specific patterns; (4) lumbar puncture with multiplex PCR for common neurotropic viruses and optional metagenomic next generation sequencing (mNGS) when needed^63^, ^64^, ^65^, ^66^; (5) virological correlation using CSF and plasma testing, including exclusion of endogenous HHV-6 (eHHV-6)^67^, ^68^; and (6) ancillary investigations such as EEG, fundoscopy, and immune profiling. Table 4 provides a parallel tabular summary of the diagnostic pathway with rationale for each step. Details of each step are provided in Supplementary File 1. This harmonised approach is intended to improve early recognition, support diagnostic consistency, and differentiate infectious from immune-mediated CNS syndromes.

**Fig. 1:**
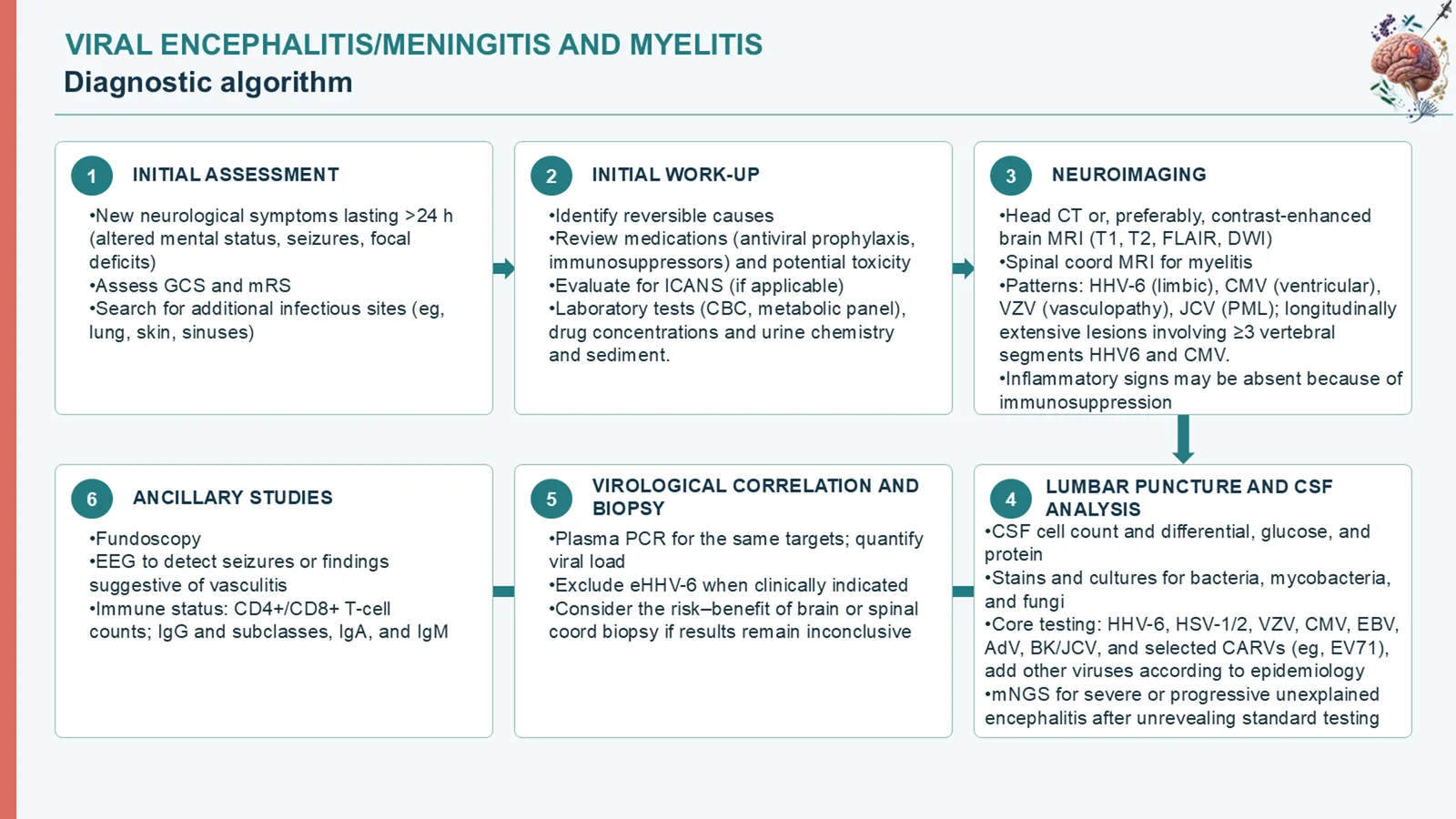
Harmonised stepwise algorithm for the diagnosis of viral encephalitis/meningitis and myelitis in HSCT and CAR T-cell recipients. The algorithm integrates clinical suspicion, MRI, CSF analysis, virological testing, and ancillary studies. A detailed breakdown of each step, including clinical thresholds, biochemical investigation, neuroimaging patterns, virological panels, and indications for advanced diagnostics (e.g., mNGS or biopsy), is provided in Table 4 and Supplementary File 1. Footnote: The proposed sequence is intended as a flexible framework. Steps may be performed concurrently without delay or reordered according to clinical urgency, patient-specific factors and local resource availability. Indicated empirical therapy should not be delayed. Abbreviations: AdV, adenovirus; BK, BK virus; CARV, community-acquired respiratory virus; CBC, complete blood count; CD4+/CD8+, CD4-positive/CD8-positive T cells; eHHV6, endogenous HHV-6; CMV, cytomegalovirus; CSF, cerebrospinal fluid; CT, computed tomography; DWI, diffusion-weighted imaging; EBV, Epstein–Barr virus; EEG, electroencephalography; EV71, enterovirus 71; FLAIR, fluid-attenuated inversion recovery; GCS, Glasgow Coma Scale; HHV-6, human herpesvirus 6; HSV-1/2, herpes simplex virus 1/2; ICANS, immune effector cell-associated neurotoxicity syndrome; IgA, immunoglobulin A; IgG, immunoglobulin G; IgM, immunoglobulin M; JCV, JC polyomavirus; mNGS, metagenomic next-generation sequencing; MRI, magnetic resonance imaging; mRS, modified Rankin Scale; PML, progressive multifocal leukoencephalopathy; T1, T1-weighted; T2, T2-weighted; VZV, varicella-zoster virus.

**Table 4:** Stepwise harmonised diagnostic approach for viral encephalitis after HCT or CAR-T therapy.

Step	Diagnostic procedures	Actions and key considerations
1. Clinical suspicion^a^	New neurological symptoms >24 h (altered mental status, seizures, focal deficits) Physical examination	Evaluate GCS, mRS; exclude ICANS, drug toxicity, metabolic causes Early recognition is critical; avoid diagnostic delay: consider empirical therapy. Treatment must not await MRI or LP if delayed.
2. Initial work-up	• Ongoing prophylaxis Laboratory analyses: • Complete blood count, inflammatory markers, routine parameters such as natrium, calcium, magnesium, TSH, Glucose, ammonia, urine chemistry and sediment. • Blood level of azoles, if ongoing prophylaxis or therapy, • review other medications and their levels when relevant	Identify reversible causes. Establish pre-test probability of HSV/VSV infection based on effective prophylaxis
3. Neuroimaging	MRI with T1, T2, FLAIR, DWI ± contrast	Characteristic patterns: HHV-6 (limbic), CMV (ventricular), VZV (vasculopathy), JC (PML) MRI sensitivity >90% in advanced disease; repeat if early negative
4. Lumbar puncture and CSF analysis	Cell count, protein, glucose, Gram stain and culture, PCR/NGS for common viruses Core CSF testing^b^ includes HHV-6, HSV-1/2, VZV, CMV, EBV, AdV, BK/JC, and some CARVs such as EV71 CARVs. Other viruses should be included according to epidemiology, syndrome, MRI, timing and systemic findings.^d^	Early CSF testing is critical but initial CSF could be normal. Repeat PCR only when early sampling, persistent high suspicion, uncertain initial result or progression could change management. Use mNGS for severe/progressive unexplained encephalitis after unrevealing conventional testing.
5. Virological correlation	Plasma PCRs for same targets; quantify viral load^c^, considered the risk/benefit of brain biopsy if inconclusive results	Exclude eHHV6 if clinically indicate Parallel systemic monitoring improves specificity although plasma/blood PCR does not substitute CSF PCR
6. Ancillary tests	EEG Fundoscopy Iimmune reconstitution (CD4/CD8, IgG)	Detect seizures, vasculitis, intracranial hyopertension, and immune deficiency

Abbreviations: MRI, magnetic resonance imaging; CSF, cerebrospinal fluid; PCR, polymerase chain reaction; NGS, next-generation sequencing; eHHV6, endogenous HHV-6; ICANS, immune effector cell-associated neurotoxicity syndrome; mRS, modified Rankin scale; GCS, Glasgow Coma Scale; HSV, herpes simplex virus; VZV, varicella-zoster virus; HHV-6, human herpesvirus 6; CMV, cytomegalovirus; EBV, Epstein–Barr virus; AdV, adenovirus; JC, JC virus; CARVs, community acquired respiratory viruses.

a The initial assessment should include a detailed clinical history covering the type and timing of HCT or CAR-T-cell therapy, donor and recipient viral serostatus when informative and available, immune status, GVHD, current and previous immunosuppressive and antiviral treatment, and recent or previous episodes of viral DNAemia or systemic viral disease. Medication and vaccination history, recent infections or infectious contacts, travel and mosquito, tick or other relevant vector exposures should also be reviewed. Physical examination should include a comprehensive neurological assessment and evaluation for extra-neurological findings that may suggest a specific viral etiology, including skin or mucosal lesions, respiratory manifestations, retinitis or other end-organ involvement. Serostatus and blood DNAemia may support risk assessment but cannot independently confirm or exclude viral CNS infection.

b Viral targets vary by platform: BioFire FilmArray ME includes HSV-1/2, VZV, CMV, HHV-6, enterovirus and parechovirus; QIAstat-Dx includes the same targets except CMV; and Allplex additionally covers EBV, HHV-7, adenovirus, mumps and parvovirus B19. Positive HHV-6, CMV or EBV results require dedicated quantitative CSF and blood PCR; eHHV6 should be excluded when appropriate. Negative panels require targeted testing for omitted pathogens, including JCV/BKV, and enterovirus typing when myelitis is suspected.

c Blood PCR complements evidence of replication but cannot confirm or exclude CNS disease: positivity may reflect unrelated reactivation, whereas negativity does not exclude CNS-confined infection. Interpretation depends on viruses, timing, specimens, and assay; serum, plasma, and whole-blood loads are neither interchangeable nor comparable across laboratories. Integrate results with clinical, MRI, and CSF findings. Serial testing should use identical specimens and assays; persistently high HHV-6 DNA warrants evaluation for endogenous HHV-6.

d Arboviruses should be investigated when suggested by travel or residence, season, local circulation, and mosquito, tick, or other vector exposure. Testing should be geographically directed and may include West Nile virus, tick-borne encephalitis virus and other regionally relevant arboviruses. Because viremia may be transient and PCR sensitivity in blood or CSF may be limited at neurological presentation, virus-specific IgM testing in serum and CSF, supplemented by paired or confirmatory serology when appropriate, should be considered. Serological findings require cautious interpretation because immune responses may be delayed or absent in HCT and CAR-T recipients and cross-reactivity may occur among flaviviruses.

## Treatment

### General principles

The management of viral encephalitis/meningitis after allogeneic HSCT relies on rapid initiation of empirical or targeted antiviral therapy, reduction of immunosuppression when feasible, and appropriate neurological supportive care and virological monitoring. Because mortality and long-term neurological sequelae risk are high,^11^, ^17^, ^69^, ^70^ particularly for HHV-6, HSV/VZV and CMV encephalitis, early empiric treatment is justified when clinical and radiologic findings are compatible and virological results are pending. Finally, adoptive transfer of virus-specific T cells (VST) shows promise but remains limited to specialised centres.^71^ Several reviews and guidelines have addressed initial workup and empirical therapy in suspected viral encephalitis.^72^, ^73^, ^74^, ^75^ Empirical antiviral treatment should be selected according to the relative pre-test probability of HHV-6/CMV versus HSV/VZV as summarised in Table 5. Foscarnet is preferred when HHV-6 is more likely, particularly during the early post-allogeneic HSCT period in profoundly immunosuppressed patients receiving reliably administered acyclovir prophylaxis, or when acyclovir-resistant HSV is suspected. An alternative option for acyclovir-resistant HSV is an helicase-primase inhibitor currently available on compassionate use in Europe. Intravenous acyclovir is preferred when sensitive HSV/VZV is more likely, particularly after antiviral prophylaxis has been discontinued, interrupted, or inadequately administered, and during later post-transplant periods when HHV-6 encephalitis is less frequent. Treatment selection should also consider renal function, cytopenias, electrolyte abnormalities, and previous antiviral exposure.

**Table 5:** Empirical antiviral therapy algorithm summary.

Step/clinical scenario	Recommended approach	Rationale	References
Step 0 — before branching	Urgent contrast-enhanced brain MRI and lumbar puncture with cell count, protein, glucose, and quantitative multiplex CSF PCR (HHV–6B, HSV-1/2, VZV, CMV, EBV, JCV/BKV, adenovirus, enterovirus); EEG if seizures suspected or unexplained altered consciousness; serum sodium; early infectious diseases and neurology consultation	CSF PCR is the diagnostic cornerstone	72, 73
Suspected encephalitis/meningitis during early post–allo-HCT (weeks 2–6), intense immunosuppression, limbic phenotype and/or preceding SIADH	Empiric foscarnet 90 mg/kg q12h or 60 mg/kg q8h (180 mg/kg/day); do not wait for CSF PCR	HHV-6B is the leading cause of viral encephalitis in this window; foscarnet also covers HSV/VZV, including TK-mutant strains	74, 75
Encephalitis breaking through correctly administered acyclovir prophylaxis	Empiric foscarnet is the preferred option	Acyclovir has no activity against HHV-6; breakthrough HSV/VZV raises concern for TK-mediated resistance, for which foscarnet is the agent of choice	74, 75
Prophylaxis interrupted/malabsorption, late post-HCT period, or phenotype highly suggestive of HSV/VZV	IV acyclovir 10 mg/kg q8h, consider to add foscarnet if HHV-6 or CMV remain plausible	Empiric acyclovir in all suspected encephalitis is an IDSA level-A recommendation	72, 75
Foscarnet contraindicated (renal impairment, refractory electrolyte disturbance)	IV acyclovir or Ganciclovir 5 mg/kg q12h according to the most probable etiology	Agent choice should be driven by myelosuppression (ganciclovir) versus nephrotoxicity (foscarnet) risk; acyclovir retained for adequate HSV/VZV coverage	^74^
Severe cytopenias or pre-engraftment	Prefer IV foscarnet or acyclovir according to the most probable etiology	Ganciclovir myelosuppression; combination therapy before engraftment has been associated with worse survival	^74^

Abbreviations: MRI, magnetic resonance imaging; CSF, cerebrospinal fluid; PCR, polymerase chain reaction; HSV, herpes simplex virus; VZV, varicella-zoster virus; HHV-6, human herpesvirus 6; CMV, cytomegalovirus; EBV, Epstein–Barr virus; AdV, adenovirus; JC, JC virus; CARVs, community acquired respiratory viruses; EEG, electroencephalogram; allo-HCT, allogeneic stem cell transplantation; TK, tyrosine kinase; SIADH, syndrome of inappropriate antidiuretic hormone secretion; IDSA, Infectious Diseases Society of America; ASTCT, American Stem Cell Transplantation and Cellular Therapy Society.

### Therapy by pathogen

Treatment of viral encephalitis in allogeneic HSCT recipients should be guided by viral etiology, CNS penetration, and patient-specific immune status. Supplementary Table S1 outlines key antiviral agents used in the targeted treatment of CNS viral infections, highlighting their dosing, viral spectrum, CNS penetration, available human pharmacokinetic data, and recommended therapy duration. Therapy should be initiated promptly, adjusted for renal function, and continued for at least three weeks or until virological clearance in blood and/or CSF is documented.

HHV-6 and CMV encephalitis are treated with intravenous foscarnet or ganciclovir; combination therapy may be considered in severe or refractory cases, though with higher toxicity risk.^76^, ^77^, ^78^ Maribavir and letermovir are not recommended due to poor CNS penetration.^56^ CMV-specific T-cell therapy may be considered when available.

HSV-1/2 and VZV are managed with intravenous acyclovir. In VZV vasculopathy, corticosteroids (e.g., methylprednisolone 1 mg/kg/day) for 5–7 days may be added.^5^ In suspected acyclovir resistance, foscarnet or a new helicase-primase inhibitor (pritelivir or amenamivir) can be considered as compassionate use.

EBV encephalitis and CNS-PTLD are distinct entities that cannot be differentiated by CSF EBV DNA alone; CNS-PTLD requires integrated imaging, CSF, staging, and, when feasible, histopathological assessment. Non-PTLD EBV encephalitis has no proven pathogen-directed therapy; management is supportive, with cautious immunosuppression reduction, while antivirals lack demonstrated efficacy and EBV-specific VSTs remain investigational. CNS-PTLD is treated with rituximab, alone or combined with chemotherapy, VST, or radiotherapy.^79^ In a pilot study, tabelecleucel induced a high response rate of 78% and demonstrated promising survival (70.6% and 54.9% at one and 2 years after VST therapy, respectively) among patients with refractory EBV + CNS PTLD.^80^ Corticosteroids may help in immune-mediated cases. Antivirals are generally ineffective.^81^, ^82^

Adenovirus encephalitis has no established CNS-directed therapy. Reduction of immunosuppression and immune recovery should be pursued whenever feasible. Cidofovir or adenovirus VSTs (if available) may be considered off-label, particularly in disseminated disease or concurrent significant DNAemia, but evidence for CNS efficacy is anecdotal and its use requires careful renal monitoring. Brincidofovir is a potential future option with a better CNS profile.^83^

No antiviral treatment of established efficacy is available for JC polyomavirus-associated PML or BK polyomavirus encephalitis. Management should prioritise immune reconstitution. Cidofovir and mirtazapine^84^ lack demonstrated efficacy and should not be recommended as routine therapy. Checkpoint inhibitors and polyomavirus VSTs remain investigational and should be restricted to clinical trials or expert-center programs.^85^, ^86^

For other viruses, supportive care is key. Ribavirin or neuraminidase inhibitors (e.g., oseltamivir) may be considered in specific cases (e.g., RSV, influenza). Immunoglobulins may help in EV71-related encephalitis.

## Harmonised response assessment and response definition

Response assessment should be conducted by a multidisciplinary team (hematology, cell therapy, infectious diseases, neurology, neuroradiology specialists) and documented either at the end of the planned treatment course or upon clinical deterioration. Importantly, the timing and approach to response evaluation may vary depending on the viral etiology and the type of therapy administered. For instance, in HHV-6 encephalitis, early follow-up (within 2–3 weeks after antiviral therapy onset) with brain MRI and/or assessment of CSF or plasma viral load kinetics may provide valuable insights into therapeutic efficacy.^87^ In contrast, JC virus-related PML typically follows a subacute course, where radiological changes may lag behind clinical progression, and treatment relies primarily on immune reconstitution. In such cases, response assessment may require longer follow-up intervals (e.g., 4–8 weeks). Treatment response in viral encephalitis/meningitis is summarised in Table 6. Response can be evaluated across clinical, virological, and radiological domains. While optimal management relies on integrating all three, in patients with persistent neurological sequelae, virological clearance and radiological stabilisation or improvement may serve as more objective indicators of treatment response. Repeating CSF PCR and MRI at 2–8 weeks or at the end of planned therapy should be considered to assess virological and radiological response and guide decisions on treatment changes or discontinuation in cases with persistent or progressive neurological symptoms. Viral load monitoring in CSF by quantitative PCR appears to be useful in the assessment of the response to treatment in CMV encephalitis^88^ and in HSV-1 encephalitis.^89^, ^90^ A follow-up MRI at 1–3 months after a first evaluation could be considered in cases with neurological sequelae.

**Table 6:** Summary of response definitions.

Response Category	Definition
Complete Response (CR)^a^	W/O neurological sequelae: Complete clinical resolution without sequelae irrespective of virological and/or radiology assessment.
	With neurological sequelae: Cases with persistent (>4 weeks) clinical improvement or stable residual neurological deficits (sequalae^c^), resolution of MRI lesions, EEG abnormalities and negative viral PCR results in CSF^b^ and/or plasma ≥7 days apart.
Partial Response (PR)^d^	Cases with clinical improvement or stable residual neurological deficits or functional scores improvement (e.g., mRS or GCS) and ≥1-log_10_ viral load reduction in CSF^b^ and/or plasma, and radiologic improvement without new lesions.
Stable disease	Cases not reaching CR or PR criteria at the time of response assessment, stable MRI lesions, no viral load change or reduction < 1-log_10_ in CSF^b^ and/or plasma
Progressive Disease (S/P D)	Progressive neurological symptoms at the time of response assessment, progressive MRI lesions, no viral load change or reduction <1-log_10_ viral load in CSF^b^ and/or plasma.
Death	Attribution to VE/VM should be reported

Abbreviations: MRI, magnetic resonance imaging; EEG, electroencephalogram; CSF, cerebrospinal fluid; mRS, modified Rankin scale; GCS, Glasgow Coma Scale.

a The two CR definition categories represent alternative, not cumulative, pathways. Complete clinical recovery without sequelae requires no ancillary confirmation, whereas in patients with stable residual neurological deficits, resolution of active infection should be supported by radiological and/or virological resolution.

b Repeat lumbar puncture is not recommended after complete clinical resolution (except for CMV and HSV). It may be considered, when safe, in patients with persistent neurological deficits if CSF findings are expected to guide treatment discontinuation or modification because of suspected treatment failure or antiviral resistance.

c The presence or absence of neurological sequelae should be reported separately from the overall response assessment since there are no associations between the length of therapy and the probability of experiencing improvement of neurological sequelae.

d In patients with persistent but stable or improving neurological manifestations, repeat lumbar puncture may be considered, when safe, to distinguish ongoing CNS infection from residual sequelae and to support treatment discontinuation when criteria for complete response with sequelae are otherwise fulfilled.

## Outcomes and prognosis

### General overview

Outcomes of viral encephalitis/meningitis following allogeneic HSCT or CAR T-cell therapy remain poor, with high mortality. The prognosis varies considerably by viral etiology, timing of onset, immune reconstitution status, and promptness of diagnosis and therapy.

### Mortality and survival

Across published cohorts, the overall case-fatality rate for viral encephalitis after allogeneic HSCT ranges between 30% and 60%.^54^, ^91^ In the IDWP study of 97 HHV-6 patients, 30-day mortality was 27%, and overall survival at 6 months was 62%, with deaths primarily due to neurological progression or concurrent infections.^11^ In another multicenter study including 32 cases of post-transplant viral encephalitis mortality reached 53% despite antiviral therapy, emphasising the poor prognosis associated with delayed diagnosis or disseminated infection.^13^ Mortality in CMV or adenovirus encephalitis is similarly high (40–70%), while HSV and VZV encephalitis, if treated promptly, have lower mortality rates (<20%).^15^, ^16^, ^92^ In CAR T-cell recipients, mortality and long-term neurocognitive outcomes appear comparable when a viral etiology is confirmed.^28^, ^29^, ^30^, ^31^, ^93^, ^94^

### Predictors of outcome

Reported conditions associated with adverse outcomes include: allogeneic-HSCT related factors, such as non-myeloablative conditioning,^11^ use of cord-blood or haploidentical grafts, reflecting delayed immune reconstitution,^14^, ^44^ ongoing severe GVHD or high-dose corticosteroid use^14^ and/or profound lymphopenia^95^; and viral encephalitis and treatment related factors, such as non-HSV viral encephalitis,^13^ multifocal MRI involvement or brainstem/ventricular extension,^54^ high CSF or plasma viral load at diagnosis,^14^ and/or delayed initiation of antiviral therapy (>1.5 days after onset).^44^

A recent study developed the GTM (abbreviation for a model composed of three risk factors: GCS score <9 [2.5 points], severe thrombocytopenia [platelet count<20,000 per μL; 1 point], and lesions >3 lobes on MRI [2.5 points]) score to predict outcomes in allogeneic HSCT recipients with viral encephalitis, based on three variables: patients in the low-risk group (0–1 points) had an overall mortality of 11%, whereas those in the high-risk group (2.5–6 points) showed a 6-month mortality of 80%.^54^ This unvalidated score may inform future risk-stratification research but should not guide routine management.

### Neurological sequelae and long-term recovery and quality of life

Long-term follow-up studies are scarce. Among viral encephalitis survivors, persistent neurological deficits occur in 30–40% of cases.^70^ The most frequent sequelae include chronic neurocognitive impairment, anterograde amnesia, concentration deficits, emotional lability, executive dysfunction, epilepsy, and psychiatric symptoms consistent with limbic or temporal lobe injury. HHV-6–associated limbic encephalitis commonly results in selective hippocampal damage, detectable on MRI as atrophy or gliosis. Patients with delayed antiviral therapy, prolonged viremia, or extensive bilateral MRI lesions are at the highest risk for irreversible cognitive decline. In contrast, early antiviral initiation, particularly within 48 h of symptom onset, correlates with significantly improved neurological recovery.^92^ Rehabilitation interventions usually implemented after head trauma injury might be effective for viral encephalitis patients.^96^ Neuropsychological rehabilitation and seizure prophylaxis are recommended components of post-recovery care.

MRI follow-up at 3-months and 12-months after treatment typically shows hippocampal atrophy or leukoencephalomalacia in cases of HHV-6 and CMV viral encephalitis, correlating with cognitive outcomes. Despite these sequelae, early recognition and structured multidisciplinary management have progressively improved functional recovery and quality of life in recent years.

## Secondary prophylaxis and pre-emptive strategies after viral encephalitis/meningitis

Preventive strategies focus on viral reactivation surveillance, antiviral prophylaxis where indicated, and optimising immune reconstitution after viral encephalitis.

HSV and VZV secondary prophylaxis with acyclovir or valacyclovir should be considered for at least 6–12 months after resolution of viral encephalitis, provided that the patient is no longer receiving immunosuppression and has no active GVHD. Discontinuation of prophylaxis should also consider evidence of immune reconstitution, such as rising CD4+ T-cell counts, recovery of immunoglobulin levels, documented vaccine responses, and complete resolution of GVHD-related signs or symptoms.

Based on expert opinion, CMV DNA PCR-based monitoring and prevention, with secondary letermovir prophylaxis, could be considered in high-risk patients after CMV viral encephalitis resolution for at least 3 months and/or until CMV-specific T-cell restoration. Regular plasma CMV DNA monitoring is required, and breakthrough or increasing DNAemia should prompt active anti-CMV treatment and, whenever feasible, resistance testing, particularly for UL56 mutations. However, information on letermovir's penetration into the CNS is very limited. If letermovir is not available, consider other antiviral alternatives. Letermovir does not protect against HSV, VZV, HHV-6, or adenovirus and thus should not be relied upon as broad CNS prophylaxis.

HHV-6 secondary prophylaxis is not recommended, as routine antivirals do not prevent reactivation, and viremia monitoring has not consistently predicted encephalitis.

For EBV, consider extending PCR monitoring of EBV DNAemia after EBV viral encephalitis resolution. Patients at high risk for EBV-PTLD after allogeneic HSCT should be closely monitored for symptoms or signs attributable to PTLD or other end-organ EBV disease. No recommendations exist for secondary pharmacological prevention of EBV infection.

Secondary prophylaxis is not recommended for adenovirus, BK, and JC viruses.

CARVs should be prevented through annual influenza, COVID-19, and possibly RSV vaccination, whereas respiratory isolation measures should be implemented, in particular during outbreaks.

Finally, restoring antiviral T-cell immunity by reducing or withdrawing immunosuppression and/or adoptive VST therapy likely remains the most effective long-term preventive measure, particularly for patients with prolonged lymphopenia or recurrent viral reactivation, and should be explored in prospective clinical trials.

## Viral myelitis: a focused clinical overview

Viral myelitis represents a rare but recognised manifestation of neurotropic viral infections in allogeneic HSCT recipients and, more rarely, after CAR T-cell therapy. Although considerably less frequent than viral encephalitis,^22^, ^23^, ^24^ it warrants specific diagnostic and therapeutic consideration due to its distinct anatomical involvement, clinical presentation, and outcome profile. Evidence on viral myelitis after HSCT and CAR T-cell-cell therapy is limited to isolated reports and small series; therefore, we provide a focused clinical overview.

### Definition, differential diagnosis and classification

Myelitis may arise from either infectious or non-infectious mechanisms. The differential diagnosis should include autoimmune or immune-mediated demyelinating disorders,^97^, ^98^, ^99^ such as multiple sclerosis, neuromyelitis optica spectrum disorder and acute disseminated encephalomyelitis; compressive spinal lesions; spinal epidural infection; vascular myelopathy; relapse or malignant involvement of the spinal cord or leptomeninges; nutritional deficiencies; drug-related neurotoxicity; and other transplant-related complications, including chronic GVHD-associated CNS inflammation.^100^

Viral myelitis is defined as inflammation of the spinal cord resulting from direct or immune-mediated viral injury. Infectious etiologies include HHV-6, CMV, VZV, human parvovirus B19 (B19V), and, in isolated reports, adenovirus, HSV and enteroviruses.^22^, ^23^, ^101^, ^102^ Myelitis might be classified as proven/probable (see Table 3) based on the pattern, e.g., by clinical presentation, neuroimaging, and other diagnostic findings. These patterns may include, among others, transverse myelitis, longitudinally extensive transverse myelitis (LETM), myeloradiculitis, and acute flaccid myelitis (AFM).

### Epidemiology and viral aetiology

The overall incidence of viral myelitis after allogeneic HSCT is very low, and published data are limited to small case series. HHV-6 accounts for most reported cases, typically occurring within the first 60 days after transplantation and frequently in cord-blood or haploidentical recipients.^22^, ^23^, ^24^ VZV, CMV, and Parvovirus B19 are less frequent but well-documented etiologic agents.^23^, ^99^, ^101^

### Clinical presentation

Myelitis can be associated with a wide range of clinical symptoms, including gait disturbances, weakness, sensory deficit, and impaired bladder and bowel control. Pruritus, pain, or numbness have been identified as important clinical symptoms in a retrospective analysis of 19 allogeneic HSCT recipients with HHV-6 myelitis.^22^ The course can be acute or subacute, and relapsing cases have been described in association with autoimmune or GVHD-related inflammation.^100^

### Diagnosis and management

Diagnosis relies on spinal MRI and CSF analysis. MRI typically demonstrates T2-weighted hyperintensity within the cervical or thoracic cord; longitudinally extensive lesions involving ≥3 vertebral segments (LETM) are frequent in HHV-6 or CMV infection.^22^, ^23^, ^24^ CSF studies should include cell count, protein, glucose, however, normal CSF parameters do not exclude disease. Molecular testing by multiplex PCR or NGS panels, such as the FilmArray Meningitis/Encephalitis Panel (FAMEP), should be considered.^101^, ^103^ Although rapid, this panel omits EBV, adenovirus, JC/BK polyomaviruses, and parvovirus B19; therefore, a negative result does not exclude viral myelitis. Targeted PCR or CSF mNGS should be considered in unresolved cases. Qualitative HHV-6 or CMV results require interpretation alongside clinical findings, spinal MRI, quantitative viral load, and blood testing.

Empirical antiviral therapy as suggested for viral encephalitis is reasonable when MRI and clinical findings suggest viral infection, pending confirmation. Treatment should target the underlying etiology according to existing guidelines.^103^ HHV-6, CMV, and VZV should follow the same antiviral and dosing principles as for viral encephalitis. VZV myelitis may require corticosteroids if vasculitis is suspected. Parvovirus B19 myelitis may respond to high-dose intravenous immunoglobulin.

### Secondary prophylaxis, prevention, and prognosis

No specific prophylaxis against viral myelitis is established, although we suggest following the same recommendations as for viral encephalitis. Vaccination remains essential: a recent study found that poliomyelitis vaccination initiated during the first year after allograft is immunogenic.^104^ Therefore, it should be performed in every HSCT recipient.

Reported outcomes of viral myelitis vary. HHV-6 myelitis generally has a favourable prognosis with early antiviral therapy, although residual sensory or sphincter dysfunction can persist.^22^, ^23^, ^24^

## Conclusions

When viral encephalitis/meningitis or myelitis is suspected, the initial diagnostic investigation should include CSF PCR for HSV 1-2, VZV, EBV, CMV, HHV6, adenovirus, and JC virus. The likelihood that encephalitis-meningitis/myelitis is caused by a virus should be classified according to probable/proven category. Empirical antiviral therapy with acyclovir or foscarnet should be started immediately in patients with strong clinical and MRI suspicion of viral encephalitis/meningitis or myelitis. The choice of first line empiric antiviral drug should be based on the pre-test probability of HHV6, HSV, VZV or CMV.

The viral encephalitis/meningitis or myelitis treatment response should be evaluated at 2–8 weeks after therapy onset using standardised EBMT categories (Complete response, partial response, stable disease, or progression). Supportive care measures in probable/proven viral encephalitis/meningitis or myelitis should include reduction of immunosuppression whenever feasible, while carefully balancing the risk of GVHD.

## Contributors

All co-authors conceptualised the subject for this workshop; participated in the f/f meeting and wrote the original manuscript draft; and played an important role in interpreting results, revised the manuscript, approved the final version, and agreed to be accountable for all aspects of the work in ensuring that questions related to the accuracy or integrity of any part of the work are appropriately investigated and resolved. They also accepted final responsibility for the decision to submit for publication. JLP, JS, SC, and PL performed the literature searches, while all panel members participated in evidence appraisal and final expert consensus. All authors read and approved the final version of the manuscript.

## Declaration of interests

Conflicts of interest have been declared by all group members and are listed below. None declared conflicts related to this work.

JLP: Advisory support: MSD; AstraZeneca; GSK; Pfizer; Moderna. Support for meeting attendance: MSD, Pierre Fabre. SC: Declares no conflicts of interest related to this work.

JS: MSD, AstraZeneca, Sanofi, Gilead. Support for meeting attendance: MSD, AstraZeneca, Roche, Gilead. Advisory Board: MSD, AstraZeneca. Declares no conflicts of interest related to this work.

MSH: Declares no conflicts of interest related to this work.

MM: speaker and advisory board member for Gilead, Mundipharma, Pfizer, and Shionogi, not related to this work.

Dionysios Neofytos: grants from Mundipharma and Takeda, consulting fees from Shionogi and MSD, honoraria for lectures from Gilead, MSD, and Takeda; all payments made to his institution, not related to this work.

FB: Advisory board AMGEN, not related to this work.

David Navarro: Declares no conflicts of interest related to this work.

FO: Declares no conflicts of interest related to this work.

AR: Declares no conflicts of interest related to this work.

ISO: Declares no conflicts of interest related to this work.

RdC: Declares no conflicts of interest related to this work.

DA: speaker for MSD, Shionogi and Takeda, not related to this work.

IYA: Declares no conflicts of interest related to this work.

PL: honoraria for lectures and consulting fees from MSD, Takeda, Pfizer and Symbio Pharmaceuticals. No conflict of interest related to this work.
