# Supplementary material for: Syndrome-oriented CNS infectious complications after hematopoietic stem-cell transplantation and CAR T-cell therapy, part 1—viral encephalitis, meningitis, and myelitis: recommendations from the Practice Harmonization & Guidelines Committee of the EBMT
Source: eClinicalMedicine. 2026 Nov 5;101:104312. doi: 10.1016/j.eclinm.2026.104312 (PMC13769232; doi:10.1016/j.eclinm.2026.104312)
Supplement: Supplementary file 1 [file mmc1.docx]

**SUPPLEMENTARY FILE**

Syndrome-oriented CNS infectious complications after Hematopoietic Cell Transplantation and CAR-T Therapy: Part 1. Viral encephalitis, meningitis, and myelitis. Recommendations from the Practice Harmonization & Guidelines Committee of the EBMT.

Contents:

Expanded diagnostic algorithm.

1. Clinical Suspicion and Initial Assessment
2. Neuroimaging
3. Lumbar Puncture and CSF Analysis
4. Virological Correlations
5. Ancillary studies

Table S1: Pharmacological profile of Antivirals Used for Viral Encephalitis: Spectrum, Dosing, CNS Penetration, Clinical Data and Therapy Duration.

Additional references

References to Table S1

1) **Expanded Diagnostic Algorithm for Viral Encephalitis in Allo-HCT and CAR-T Recipients**

This supplementary material expands upon the five-step diagnostic algorithm summarized in Figure 1 and Table 4, providing detailed technical guidance and rationale for each diagnostic action.

**Step 1. Clinical Suspicion and Initial Assessment**

- **Indications:** Any allo-HCT recipient presenting neurological syndrome or CAR-T cell recipients with unexpected ICANS evolution/behavior.
- **Immediate actions:**
  - Neurological examination (Glasgow Coma Scale, GCS), modified Rankin Scale (mRS), fundus, complete physical examination and vital signs (fever, oxygen levels, heartbeat, respiratory frequency, and arterial pressure).
  - Lab tests (cell blood counts, metabolic panel (including glucose, acid-base balance, ammonia, electrolytes, blood gases, lactate, LDH, plasma osmolarity, coagulation profile, serum vitamin B1), liver/renal function, thyroid function, inflammatory markers (CRP, ferritin, fibrinogen).
  - Critical review of medications and assessment of drug levels or doses where relevant for neurotoxicity^64^ (such as calcineurin inhibitors, triazoles, fludarabine, cytarabine, busulfan, and several other drugs).
  - Evaluate for ICANS (CAR-T context) and rule out metabolic causes.

**Rationale:** Early clinical recognition and exclusion of reversible metabolic or drug-induced etiologies prevent diagnostic delay.

**Step 2. Neuroimaging**

**Preferred modality:** Brain MRI with T1, T2, FLAIR, DWI, and post-contrast sequences. MRI typically demonstrates pathogen-specific but non-pathognomonic features^58,65^. It should be acknowledged, however, that MRI could be normal in >30% of infectious encephalitis^59^.

- **Characteristic radiological findings:**
  - HSV 1-2: Temporal lobe abnormalities, such as edema, hemorrhage, or necrosis in >80% of cases^66^.
  - **HHV-6:** Bilateral T2/FLAIR hyperintensities in medial temporal lobe or hippocampal hyperintensity (limbic pattern) in 60% of cases^67^.
  - **CMV:** Typical periventricular or ependymal enhancement (ventriculoencephalitis) in one third of cases^68^.
  - **EBV:** Multifocal areas in the deep gray nuclei and sub-cortical white matter in two thirds of cases^69^.
  - **VZV:** Multifocal ischemic lesions due to vasculopathy particularly at gray-white matter junctions in >90% of cases^70^. Angiography revealed abnormalities in 70% of subjects, with large and small arteries involved in 50%, with small arteries only in 37% and large arteries only in 13%^70^.
  - **AdV:** White matter or basal ganglia lesions.
  - **JC virus:** Multifocal asymmetric white matter lesions (PML pattern) in almost all cases^44^.
- **If MRI unavailable:** Non-contrast CT to exclude hemorrhage or mass lesion especially in setting of the urgent clinical assessment for a sudden neurological syndrome.

**Note:** Early MRI may be normal; repeat imaging is recommended if clinical suspicion persists^58,71^.

**Step 3. Lumbar Puncture and CSF Analysis**

- Perform when not contraindicated by coagulation and platelets parameters and once space-occupying lesions and intracranial hypertension are excluded.
- **CSF studies:**
  - Cell count, protein, glucose. Normal CSF does not exclude VE; cell counts <10/mm³ are common in HHV-6 and CMV infections^72,73^. Contamination should be excluded in samples with high RBC counts.
  - First line PCR by mono or oligoplexed PCR or by using a CSF syndromic panel should include HHV-6, HSV-1/2, VZV, CMV, EBV, AdV, BK/JC, and some CARVs such as EV71 viruses^74^. Consider extending the microbiological workup when more common pathogens are not detected and the clinical context supports further investigation^10^, for example, testing for west Nile Virus (WNV) or tick born encephalitis virus (TBEV) in endemic regions or in patients returning from areas with recent emergence due to climate-related shifts, such as Greece, Italy, or other newly identified hot spots, HHV7, or community-acquired respiratory viruses (CARVs) such as RSV, or influenza in patients with neurological deterioration and concurrent or evolving documented respiratory infection.
  - CSF culture and Gram stain (to exclude bacterial/fungal causes).
  - An initial negative/normal CSF does not exclude VE in particular when performed within the first 48h after symptoms initiation^75^. Repeating CSF PCR is appropriate after very early sampling, persistent high clinical suspicion despite an initial negative result, an equivocal initial finding or an evolution suggesting treatment failure.
  - Store residual CSF for retrospective or expanded testing.

Note: Fundoscopic exam provides a non-invasive window into the brain's vasculature and optic nerve, revealing signs of conditions like increased intracranial pressure (papilledema), infection (toxoplasma, CMV, VZV), stroke, and neurodegenerative disease.

**Step 4. Virological Correlation**

- Additional testing of blood samples can, under some circumstances, be helpful in clinical management (i.e., HHV-6, EBV, CMV, AdV, HSV, VZV), but is not helpful in making the diagnosis. Viral PCR testing in blood may provide complementary evidence of systemic replication but cannot independently confirm or exclude viral CNS disease. A positive result may represent asymptomatic or clinically unrelated reactivation, whereas a negative result does not exclude infection confined to the CNS. Interpretation depends on the virus, timing of sampling, specimen type and assay used; serum, plasma and whole-blood viral loads are not interchangeable, and results from different laboratories may not be directly comparable. Blood findings should therefore be interpreted alongside the clinical phenotype, MRI and CSF results. When monitoring viral kinetics, serial measurements should preferably use the same specimen type and assay. Persistently high HHV-6 DNA levels should prompt evaluation for endogenous HHV-6 when appropriate^76,77^.

Advanced diagnostics:

- **Metagenomic next-generation sequencing (mNGS)** on CSF for pathogen detection when first line PCR panels are negative, but clinical/radiological VE suspicion remains high, after corrections or exclusion of other plausible causes and when the clinical course does not improve or deteriorate, particularly in the context of severe immunocompromised status^60,63^.
- Consider **brain biopsy** should be reserved for selected progressive cases that remain unexplained after adequate less-invasive investigations and in which tissue diagnosis is likely to change management balancing risk/benefit ratio.

**Step 5. Ancillary Studies**

- **EEG:** May show diffuse slowing or epileptiform activity; helpful for subclinical seizures or limbic involvement.
- **Immunological evaluation:** CD4/CD8 recovery, immunoglobulin levels, presence of GVHD or recent immunosuppressive escalation, vaccination status.

**Table S1.** Pharmacological profile of Antivirals Used for Viral Encephalitis: Spectrum, Dosing, CNS Penetration, Clinical Data and Therapy Duration.

| Antiviral | Suggested Dose (Encephalitis) | Active Against | CNS Penetration (Qualitative) | CNS Penetration (Quantitative) | Human CNS Data | Suggested Duration / Monitoring / Additional Notes  Ref 4,14-17 |
| --- | --- | --- | --- | --- | --- | --- |
| Acyclovir | 10mg/kg IV q8h;  Continuous infusion in selected difficult to treat cases (ref 19-22) | HSV-1, HSV-2, VZV | Moderate | CSF:serum AUC ratio ~0.15–0.4 | Yes | HSV encephalitis 14–21 days IV in adults and 21 days in children 3 months–12 years; neonatal CNS HSV ≥21 days with mandatory negative repeat CSF PCR at or after day 21 before discontinuation; monitor renal function and adjust dosing; virological clearance in CSF/plasma when possible |
| Valacyclovir  Ref 1-6 | 2g PO q8h (oral alternative) in adult.  Child with 3 months to 11 years: 20 mg/kg/dose (validated range 20–25 mg/kg, two to three times daily)  No licensed pediatric liquid formulation exists and that an extemporaneous suspension prepared per the label is required | HSV-1, HSV-2, VZV | Moderate | CSF:serum AUC ratio ~0.19–0.28 | Yes | Oral alternative for step-down therapy restricted to HSV meningitis in afebrile ≥48 h, improving consciousness, seizure-free, reliable enteral absorption, negative/clearing CSF PCR patients; valaciclovir is not an accepted therapy for acute HSV encephalitis |
| Famciclovir | Not routinely used for CNS infections | HSV-1, HSV-2, VZV | Limited | Scarce data | Limited | Not recommended for CNS disease due to poor penetration and limited data |
| Ganciclovir | 5 mg/kg IV q12h | CMV, HHV-6, HSV, VZV | Low to Moderate | CSF:plasma AUC ~0.15 | Yes | CMV response-guided induction of ≥3 weeks with maintenance in persistently immunosuppressed patients; monitor CBC and renal function; combination therapy possible in severe cases |
| Foscarnet | 60 mg/kg IV q8h or 90 mg/kg IV q12h | CMV, HSV, VZV, HHV-6 | Moderate | CSF:plasma AUC ~0.23–0.66 | Yes | HHV-6B ≥3 weeks and until plasma and CSF clearance (the only indication to which the original rule legitimately applied); For CMV same as ganciclovir; may be preferred in neutropenia; risk of nephrotoxicity |
| Ribavirin  Ref 7-9 | 15–20 mg/kg/day IV in three divided doses | Lassa, Arenaviruses, Hantavirus under evaluation for selected RNA viruses | Moderate | unknown | Yes | Unknow duration; Use in selected cases (e.g., EV71); monitor hemoglobin and liver enzymes |
| Cidofovir  Ref 4, 10-13 | 5 mg/kg IV weekly x2, then q2wk | CMV, AdV, BK | Poor | CSF penetration <10% | Very limited | viral-load– and immune-reconstitution–guided duration appropriate to an every-other-week schedule; Monitor for nephrotoxicity; used when other therapies fail; limited CNS data; contraindicated in renal failure;  mandatory probenecid, saline hydration, and renal contraindications; creatinine, proteinuria and ophthalmological review |

Abbreviations: HSV-1/2, herpes simplex virus type 1 or 2; VZV, varicella zoster virus; CMV, cytomegalovirus; HHV-6, human herpesvirus 6; AdV, adenovirus; BK/JC virus, BK or JC polyomavirus; CSF, cerebrospinal fluid; AUC, area under the curve; PO, per os (oral); IV, intravenous; q8h/q12h/q2wk, every 8/12 hours/every 2 weeks.

Acyclovir, ganciclovir, and foscarnet exhibit consistent CNS penetration and documented human data, while cidofovir shows poor CNS availability. Brincidofovir may have improved CNS penetration as compared to cidofovir^1^ but remains investigational. CNS penetration can be improved by optimizing both the route and infusion strategy^2^. Intravenous acyclovir achieves higher, faster and more consistent CSF levels than oral valacyclovir. In immunocompromised patients, IV administration is preferred during the acute phase. Moreover, continuous or prolonged infusions of time-dependent antivirals (e.g., acyclovir 10 mg/kg over 8–24 h) may enhance CSF exposure compared to intermittent dosing^3^. Wide interindividual variability in CNS penetration has been reported for acyclovir and ganciclovir, especially in immunocompromised hosts^4^. Although plasma drug levels do not reliably predict CSF concentrations, limiting their utility as a surrogate for CNS exposure, therapeutic drug monitoring (TDM) may be useful in selected cases of severe VE, particularly in patients with atypical pharmacokinetics, persistent infection, or suspected antiviral underexposure.

Additional references

1.	Quenelle DC, Lampert B, Collins DJ, Rice TL, Painter GR, Kern ER. Efficacy of CMX001 against herpes simplex virus infections in mice and correlations with drug distribution studies. J Infect Dis. 2010;202(10):1492-1499.

2.	Nau R, Seele J, Bodilsen J, Groß U. Optimization of antiviral dosing in Herpesviridae encephalitis: a promising approach to improve outcome? Clin Microbiol Infect. 2025 Apr;31(4):534-541. doi: 10.1016/j.cmi.2024.12.008.

3.	Sun M, Manson ML, Märtson AG, Bodilsen J, de Lange ECM, Guo T. Revisiting Acyclovir Dosing for Adult Viral Encephalitis Using a Full Bayesian LeiCNS PBPK Modeling Approach. Clin Pharmacokinet. 2025 Sep;64(9):1413-1423. doi: 10.1007/s40262-025-01545-w.

4.	Huntjens DW, Dijkstra JA, Verwiel LN, Slijkhuis M, Elbers P, Welkers MRA, et al. Optimizing Antiviral Dosing for HSV and CMV Treatment in Immunocompromised Patients. Pharmaceutics. 2023 Jan 3;15(1):163. doi: 10.3390/pharmaceutics15010163.

**References to Table S1**

Oral valaciclovir dosing:

[1]Kimberlin DW, Jacobs RF, Weller S, et al. Pharmacokinetics and safety of extemporaneously compounded valacyclovir oral suspension in pediatric patients from 1 month through 11 years of age. Clin Infect Dis. 2010;50(2):221-8. PMID: 20014952.

[2] GlaxoSmithKline. VALTREX (valacyclovir hydrochloride) [prescribing information]. Silver Spring (MD): U.S. Food and Drug Administration.

[3] Abdalla S, Briand C, Oualha M, et al. Population pharmacokinetics of intravenous and oral acyclovir and oral valacyclovir in pediatric population to optimize dosing regimens. Antimicrob Agents Chemother. 2020;64(12):e01426-20. PMID: 32988829.

[4] Panel on Opportunistic Infections in Children with and Exposed to HIV. Guidelines for the prevention and treatment of opportunistic infections in children with and exposed to HIV. Rockville (MD): Office of AIDS Research Advisory Council; 2026.

Valaciclovir step-down:

[5] Gnann JW Jr, Sköldenberg B, Hart J, et al. Herpes simplex encephalitis: lack of clinical benefit of long-term valacyclovir therapy. Clin Infect Dis. 2015;61(5):683-91. PMID: 25956891.

[6] Pouplin T, Pouplin JN, Van Toi P, et al. Valacyclovir for herpes simplex encephalitis. Antimicrob Agents Chemother. 2011;55(7):3624-6. PMID: 21576427.

Ribavirin:

[7] Kumar R, Tripathi P, Baranwal M, et al. Randomized, controlled trial of oral ribavirin for Japanese encephalitis in children in Uttar Pradesh, India. Clin Infect Dis. 2009;48(4):400-6. PMID: 19143532.

[8] Binks SNM, Saylor D, Easton A, Thakur KT, Irani SR. Encephalitis. Lancet. 2026. [9] Venkatesan A, Michael BD, Probasco JC, Geocadin RG, Solomon T. Acute encephalitis in immunocompetent adults. Lancet. 2019;393(10172):702-16.

Cidofovir:

[10] Tan CS, Koralnik IJ. Progressive multifocal leukoencephalopathy and other disorders caused by JC virus: clinical features and pathogenesis. Lancet Neurol. 2010;9(4):425-37.

[11] De Luca A, Giancola ML, Ammassari A, et al. Cidofovir added to HAART improves virological and clinical outcome in AIDS-associated progressive multifocal leukoencephalopathy. AIDS. 2000;14(14):F117-21. PMID: 11061646.

[12] Hiwarkar P, Kosulin K, Cesaro S, et al. Management of adenovirus infection in patients after haematopoietic stem cell transplantation: state-of-the-art and real-life current approach. Rev Med Virol. 2018;28(3):e1980.

[13] Carreras E, Dufour C, Mohty M, Kröger N, editors. The EBMT handbook: hematopoietic cell transplantation and cellular therapies. Cham: Springer; 2024.

Therapy duration:

[14] Tyler KL. Acute viral encephalitis. N Engl J Med. 2018;379(6):557-66.

[15] Venkatesan A, Michael BD, Probasco JC, Geocadin RG, Solomon T. Acute encephalitis in immunocompetent adults. Lancet. 2019;393(10172):702-16.

[16] Panel on Opportunistic Infections in Children with and Exposed to HIV. Guidelines for the prevention and treatment of opportunistic infections in children with and exposed to HIV. Rockville (MD): Office of AIDS Research Advisory Council; 2026.

[17] Corey L, Wald A. Maternal and neonatal herpes simplex virus infections. N Engl J Med. 2009;361(14):1376-85.

[18] Kampouri E, Handley G, Phan TL, et al. American Society for Transplantation and Cellular Therapy Series #9: management of human herpesvirus 6B after hematopoietic cell transplantation and chimeric antigen receptor T-cell therapy. Transplant Cell Ther. 2025.

Acyclovir extended infusion

[19] Continuousinfusion of high-dose acyclovir for serious herpesvirus infections.Fletcher CV, Englund JA, Bean B, Chinnock B, Brundage DM, Balfour HH Jr.Antimicrob Agents Chemother. 1989 Aug;33(8):1375-8. doi: 10.1128/AAC.33.8.1375.PMID: 2552909

[20] A continuousinfusion of acyclovir for severe hemorrhagic varicella.Kakinuma H, Itoh E.N Engl J Med. 1997 Mar 6;336(10):732-3. doi: 10.1056/NEJM199703063361016.PMID: 9045052

[21] More on continuous-infusionacyclovir for severe varicella.Krolewiecki AJ, Braffman M.N Engl J Med. 1997 Jul 17;337(3):203-4. doi: 10.1056/NEJM199707173370316.PMID: 9221354 No abstract available.

[22] Treatment of acyclovir-resistant herpes simplex virus with continuousinfusion of high-dose acyclovir in hematopoietic cell transplant patients.Kim JH, Schaenman JM, Ho DY, Brown JM.Biol Blood Marrow Transplant. 2011 Feb;17(2):259-64. doi: 10.1016/j.bbmt.2010.06.020. Epub 2010 Jul 6.PMID: 20615475
